# Estimation of Wheat Grain Protein Content at Multiple Growth Stages Based on Space–Air–Ground Collaborative Observation Data

**DOI:** 10.3390/plants15182811

**Published:** 2026-09-13

**Authors:** Mengxia Li, Junling Li, Yuchen Zhang, Haotian Ye, Ronghao Chu, Xihe Zhang, Shaoyu Han, Xian Xue

**Affiliations:** 1 CMA·Henan Key Laboratory of Agrometeorological Support and Applied Technique, Zhengzhou 450003, China; 15251718328@163.com (M.L.); zhangyc_1994@163.com (Y.Z.); 18752096177@163.com (H.Y.); ronghao_chu@163.com (R.C.); zxihe17@163.com (X.Z.); 2 Henan Institute of Meteorological Sciences, Zhengzhou 450003, China; 3 Henan Province Meteorological Data Center, Zhengzhou 450003, China; 4 Institute of Agricultural Information Technology, Henan Academy of Agricultural Sciences, Zhengzhou 450002, China; hansy@hnagri.org.cn; 5 College of Agriculture (Tree Peony), Henan University of Science and Technology, Luoyang 471023, China; xuexian2011@126.com

**Keywords:** wheat, grain protein content, unmanned aerial vehicle, scaling-up

## Abstract

Wheat grain protein content (GPC), quantified as the mass proportion of protein relative to the total dry grain weight, is a key indicator for wheat quality evaluation. Based on space–air–ground collaborative observations, this study screened GPC-sensitive spectral parameters and multi-stage and multi-scale remote-sensing GPC estimation models. Results showed that the optimal feature set derived from unmanned aerial vehicle (UAV) imagery consisted of four vegetation indices (VIs) and three optimized texture indices (TIs). The Random Forest-based Soil–Plant Analysis Development (SPAD) model for field plots achieved the highest accuracy at the anthesis stage, with a coefficient of determination (*R*^2^) of 0.91 and a root mean square error (RMSE) of 1.77. Cross-year validation gave a correlation coefficient of 0.80 and RMSE of 3.83. Data analysis indicated that GPC of mature wheat had an extremely significant correlation with SPAD values across various growth stages, higher than leaf area index (LAI). We thus established a quantitative GPC estimation framework with UAV-derived SPAD as an intermediate variable, which kept stable *R*^2^ (0.60) and RMSE (1.1%) except during grain-filling. On this basis, UAV-retrieved GPC data were scaled up to 10 m. Eight optimal parameters were selected from Sentinel-2 satellite data, and the regional-scale GPC estimation model was developed via the partial least squares regression (PLSR) algorithm. Compared with the model established directly using ground measured data without scale conversion, the scale-up model increased *R*^2^ by 8.9% and reduced RMSE by 40%, improving the model inversion accuracy. The proposed regional-scale GPC remote-sensing estimation model effectively solves the scale mismatch between ground observation and satellite data, and provides technical support for field and regional wheat quality remote-sensing estimation.

## 1. Introduction

Wheat is a major staple crop that supplies 20% of the world’s dietary protein, playing a vital role in global food security and protein provision [1]. With socioeconomic development and the advancement of agricultural supply-side structural reform, wheat supply and demand pattern have undergone marked changes, driving a sustained growth in the demand for high-quality wheat [2]. Grain protein content (GPC) serves as a core indicator for assessing wheat quality [3,4]. Rapid monitoring of wheat GPC in production can guide farmers to adopt optimized cultivation practices and implement classified harvesting and standardized grain storage. It is also of great significance to the modern management of wheat production and the realization of high yield with superior quality.

Traditional detection methods for GPC mainly include the Kjeldahl method and the sulfuric acid–hydrogen peroxide colorimetric method, which are time-consuming, labor-intensive, and costly [5]. Near-infrared and hyperspectral spectral analyses are relatively low-cost and efficient for determining GPC after wheat harvest [6]. However, similar to laboratory chemical analysis methods, they rely on discrete sampling after wheat maturation and harvesting, making it difficult to scale up to the regional scale and limiting their guidance for practical agricultural production. Remote-sensing technology, characterized by its rapidity and non-destructive detection, can efficiently acquire ground spectral information and realize the transformation of wheat GPC monitoring from point sampling to regional surface coverage, showing growing advantages in GPC estimation.

In the early stage of remote-sensing monitoring for wheat nutrition, ground-based observation platforms served as the primary tool, but were only applicable to local-scale research [7,8]. Satellites play an important role in large-scale crop monitoring, yet their applications are constrained by spatial resolution [9]. In recent years, unmanned aerial vehicles (UAVs) with high mobility and high spatial resolution have been widely adopted for monitoring crop nitrogen status, yet they suffer from limited observation coverage [10,11,12]. Direct combination of satellite data and ground observations introduces scale effects resulting from scale mismatch. Therefore, the comprehensive application of multi-source remote-sensing data can not only compensate for inherent limitations of individual data sources, but also take full use of the respective advantages of each dataset, realizing comprehensive, precise and timely monitoring of wheat GPC [13]. Various fusion techniques have been developed for integrating space–air–ground observations, which are mainly categorized into pixel-level fusion, feature-level fusion, and decision-level fusion [14,15]. Among them, pixel-level fusion has been widely applied in agricultural remote-sensing research. Nile et al. [16] adopted the spectral response function to simulate Sentinel-2A equivalent remote-sensing reflectance from UAV hyperspectral images combined with ground experimental data, enabling regional-scale inversion of leaf chlorophyll content in maize across multiple growth stages. Yao et al. [17] proposed a “coarse-fine fusion” two-step fusion method for UAV and Sentinel-2 imagery, which can mitigate artifacts caused by substantial spatial-resolution disparities compared with conventional single-step fusion approaches. In contrast to the above pixel-level studies, feature-level fusion merges multi-source feature variables rather than raw image pixels. Liu et al. [18] extracted 23 spectral features from Sentinel-2 imagery and derived three topographic features (slope, aspect, and elevation) from UAV data. These multi-source features were integrated to construct fresh-tea yield estimation models for different seasons. For decision-level fusion applications, ground data are first used to construct agronomic-parameter inversion models at the UAV scale. UAV-scale outputs are fused with satellite spectral data to enhance large-area crop-parameter estimation accuracy [19,20]. Despite the rapid progress of multi-source fusion in crop remote-sensing, most wheat GPC estimation studies still focus on modeling based on spectral information from individual platforms, and the potential of multi-source space–air–ground fusion has not been fully exploited for regional wheat GPC retrieval.

Over the past decade, extensive research has focused on remote-sensing monitoring of wheat GPC, yielding a wide range of direct and indirect estimation models. Empirical models linking remote-sensing data to GPC adopted statistical methods to quantify correlations between GPC and spectral reflectance or its derived variables, without accounting for the physiological mechanisms governing wheat grain protein formation. Existing studies have built GPC estimation models based on canopy reflectance and vegetation indices (VIs) at critical quality-determining growth stages. These models achieved favorable predictive accuracy and demonstrated the promising potential of remote-sensing for GPC monitoring [21,22]. Wheat GPC was closely associated with crop nitrogen status; variations in nitrogen nutrition altered leaf chlorophyll content and further manifested in canopy spectral reflectance [23,24]. In addition, previous studies confirm that agronomic parameters, including leaf area index (LAI) and aboveground biomass, were tightly correlated with GPC [25]. Accordingly, researchers have introduced agronomic parameters as intermediate variables to indirectly retrieve GPC data and improved the mechanistic interpretability and predictive accuracy of the models [26,27,28]. Furthermore, environmental factors exert prominent effects on wheat grain quality, with spatially heterogeneous meteorological conditions posing an especially strong impact [29,30]. Accordingly, researchers incorporated ecological variables to develop semi-mechanistic models. By integrating agronomic physiological principles and environmental drivers, these models effectively alleviated prediction biases arising from interannual and spatial extrapolation of conventional GPC monitoring models [31,32,33]. Restricted by homogeneous meteorological and ecological conditions within individual field plots, such modeling approaches are more suitable for large-area GPC monitoring. Mechanistic models coupling remote-sensing and crop models comprehensively clarified the linkage between crop growth and GPC. Using plant nitrogen accumulation as the state variable, Li et al. [34] assimilated field spectral data into the DSSAT model with particle swarm optimization to estimate winter wheat GPC, yielding a root mean square error (RMSE) of 1.16%. Different from the field scale of Li’s work, Chen et al. [35] combined Landsat imagery with the CERES-Wheat model to develop an assimilation model for regional GPC estimation. Based on two-year field experiments, Zhu et al. [36] retrieved LAI and leaf nitrogen accumulation from remote-sensing and confirmed that multivariable assimilation effectively improved GPC prediction accuracy. By comprehensively accounting for the effects of multiple drivers on crop grain quality, the model delivers stable performance across different cultivars, years and spatial sites. Nevertheless, excessive input variables lead to poor operability, and relevant research are mostly limited to plot-scale GPC monitoring.

Existing studies reveal that most previous GPC estimation research are focused on point or field scales. Regional estimation models often suffer from low accuracy and mechanistic errors caused by scale mismatch, and their practical applicability at regional scales needs to be improved. Although space–air–ground fusion provides an effective solution for resolving scale inconsistency, few studies have applied data fusion to wheat GPC inversion. Against this background, the main objectives of this study are to (1) screen out sensitive characteristic parameters, construct field-scale Soil–Plant Analysis Development (SPAD) remote-sensing estimation models based on VIs and texture indices (TIs), and further develop wheat GPC estimation models on this basis; (2) propose a scale conversion method for space–air–ground collaborative observation data to alleviate the scale mismatch between ground measured data and satellite remote-sensing data; and (3) achieve multi-scale estimation of wheat GPC at different growth stages, so as to provide theoretical and technical support for accurate wheat quality prediction and precision field management.

## 2. Results

### 2.1. Relationships Among SPAD, LAI, and GPC

**Figure 1:**
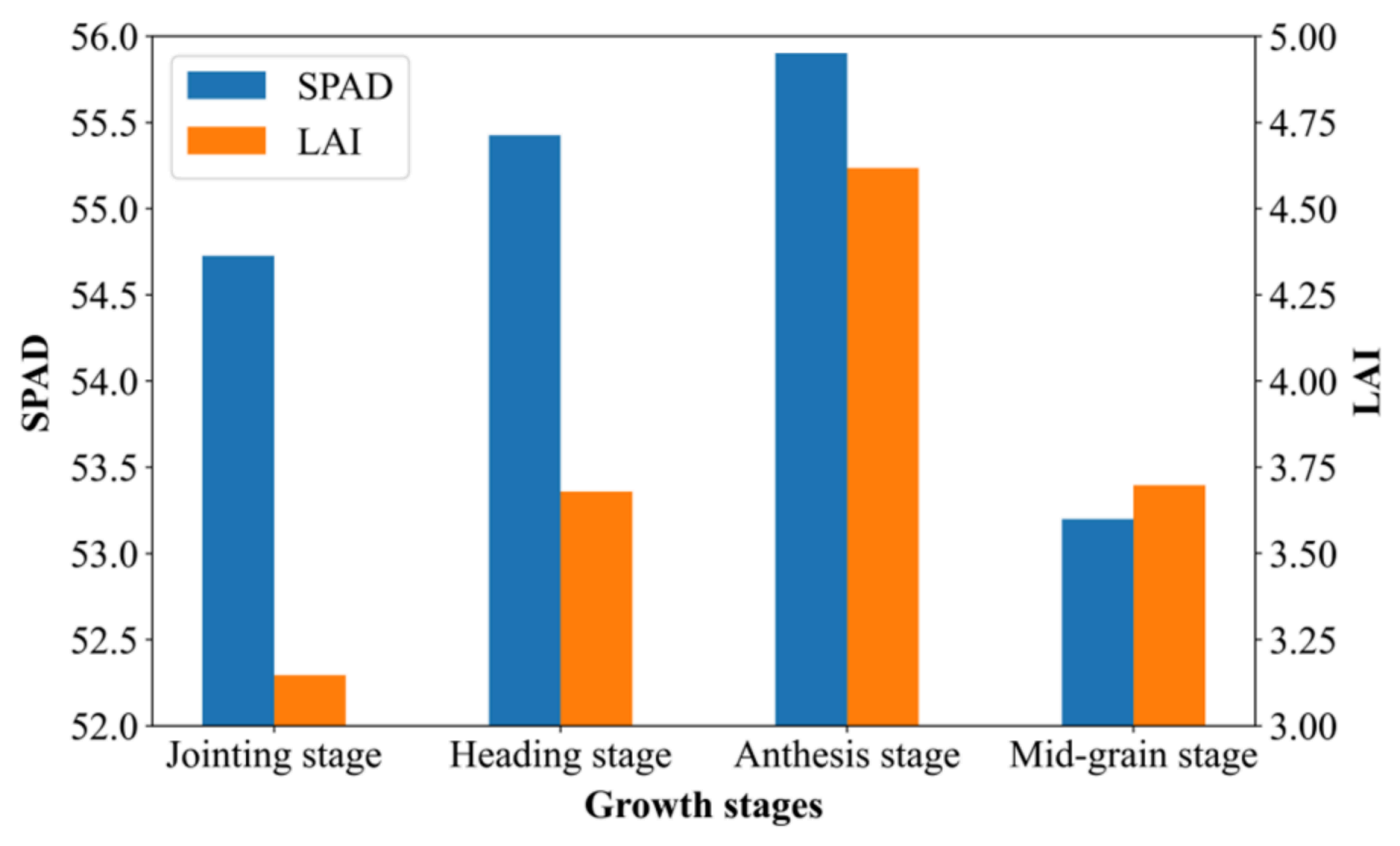
Changes in Soil–Plant Analysis Development (SPAD) values and leaf area index (LAI) of wheat at different growth stages.

**Figure 2:**
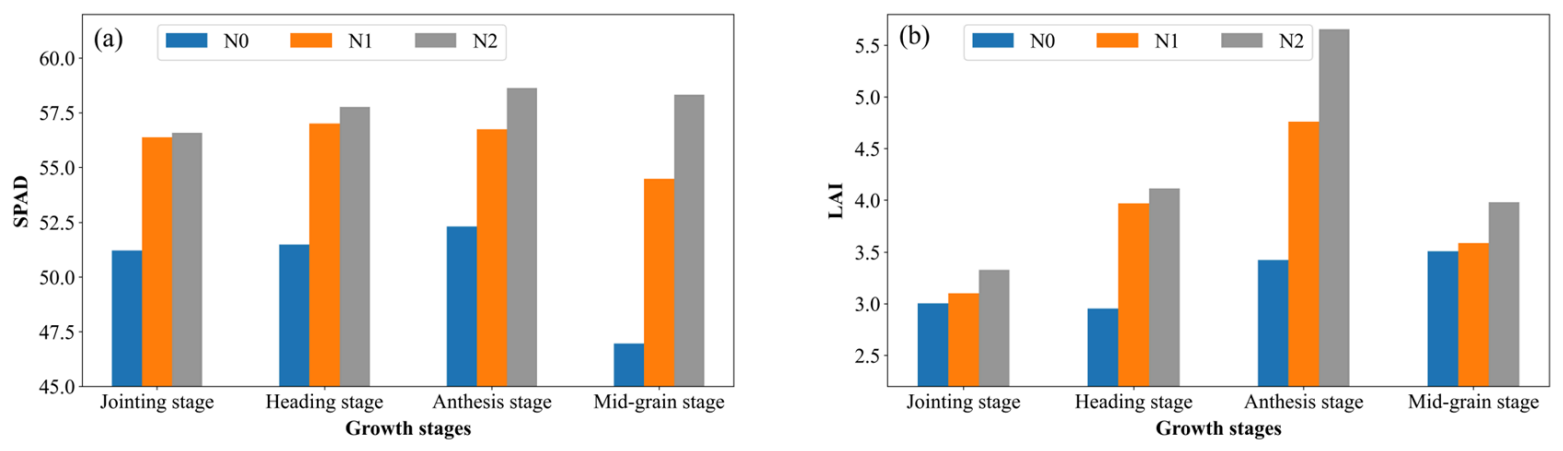
Changes in SPAD (**a**) and LAI (**b**) of wheat under different nitrogen application rates across growth stages.

As shown in Figure 1, both SPAD and LAI of wheat increased first and then decreased throughout the growth stages, with differences observed among different treatments at the same growth period. At the jointing stage, the average SPAD and LAI of wheat were 54.7 and 3.15, respectively. Both indicators peaked at the anthesis stage at 55.9 and 4.6. Afterwards, wheat shifted gradually from vegetative growth to reproductive growth, leaf growth ceased, and leaves began to senesce, leading to a decline in SPAD and LAI to 53.2 and 3.7. The effects of different nitrogen application rates on wheat SPAD and LAI across growth stages are shown in Figure 2. Under treatments N0, N1 and N2, the SPAD values were 50.5, 56.2 and 57.8, while the corresponding LAI values were 3.2, 3.9 and 4.3. Wheat under the high nitrogen rate (N2) presented the highest SPAD value and LAI at anthesis. At the mid-filling stage, the SPAD value decreased by 10.2% under the low nitrogen rate, while it remained relatively high under the high nitrogen rate, indicating strong physiological activity and environmental adaptability. Overall, the maximum values of both indicators at all growth stages were recorded under the N2 treatment. This was mainly because wheat grew rapidly from the jointing stage to the grain-filling stage, accompanied by an increased demand and uptake of nitrogen fertilizer.

Consistent with SPAD and LAI, GPC increased gradually with increasing nitrogen application rate. The GPC values under treatments N0, N1 and N2 were 9.47%, 10.31% and 10.91%, respectively. Minor differences in GPC were observed between the two wheat varieties. The average GPC was 10.13% for *Triticum aestivum* L. cv. Zhengmai 136 (hereinafter referred to as Zhengmai) and 10.33% for *Triticum aestivum* L. cv. Zhoumai 36 (hereinafter referred to as Zhoumai). Under the gradient nitrogen treatments, the GPC variation range of Zhengmai was 4.89–8.14%, while that of Zhoumai reached 13.06–22.54%. These results indicated that the GPC of Zhengmai was relatively stable under varying nitrogen supplies, whereas Zhoumai was more sensitive to nitrogen levels. Increasing nitrogen application exerted a significant promoting effect on its GPC. Under the high-nitrogen N2 treatment, the GPC of Zhengmai and Zhoumai reached 10.50% and 11.31%, respectively.

Table 1 presents the Pearson correlation coefficients between GPC of mature grains and SPAD values, as well as LAI, at different growth stages. SPAD showed an extremely significant positive correlation with GPC at all key growth stages, with the highest correlation of 0.74 at anthesis. LAI showed an extremely significant positive correlation with GPC at the heading and anthesis stages, and a significant positive correlation at the middle grain-filling stage, with weaker correlations than SPAD. In previous findings, wheat GPC was closely associated with nitrogen nutrition status. Variations in nitrogen nutrition induced changes in leaf SPAD values, which were further reflected in canopy reflectance spectra. The amount of nitrogen absorbed from soil after anthesis was related to pre-anthesis population LAI, aboveground biomass, and other agronomic traits. Accordingly, SPAD was selected as an intermediate variable linking spectral features to GPC in the later modeling stage.

High-quality GPC data were essential for accurate monitoring and prediction. Most previous studies measured GPC indirectly using laboratory near-infrared instruments, yet few validated these results against reference measurements from standard chemical analysis. In this study, GPC of wheat samples were measured using both near-infrared spectroscopy and chemical analytical methods. A high *R*^2^ (0.97) and an RMSE of 0.34% were obtained between the two approaches. This result verified that GPC values determined by near-infrared indirect measurement had excellent accuracy and reliability.

### 2.2. Field-Scale SPAD Models

#### 2.2.1. Correlation Between VIs and SPAD Values

**Figure 3:**
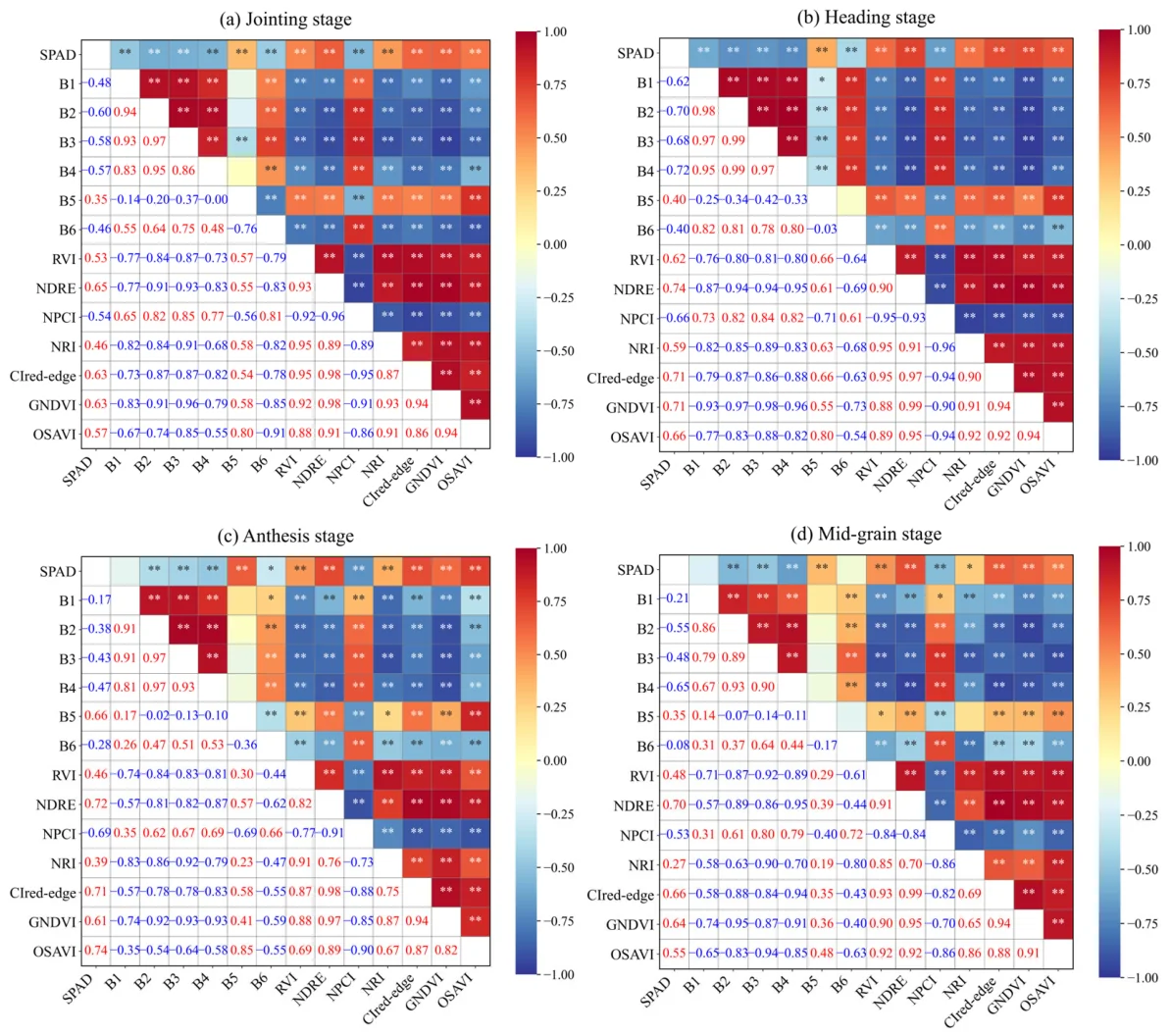
Pearson correlation coefficients of SPAD versus spectral reflectance and vegetation indices (VIs). Red numbers represent positive correlations, and blue numbers represent negative correlations. * and ** indicate significance at the 0.05 and 0.01 probability levels, respectively.

At all growth stages, SPAD values were positively correlated with reflectance in blue, green, red, red-edge and thermal infrared bands, and negatively correlated with near-infrared reflectance. The strongest correlations were observed at the jointing stage. Using UAV observation data, the VIs including ratio vegetation index (RVI), normalized difference red-edge index (NDRE), normalized pigments chlorophyll ratio index (NPCI), nitrogen reflectance index (NRI), red chlorophyll edge index (CIred-edge), green normalized difference vegetation index (GNDVI) and optimized soil-adjusted vegetation index (OSAVI) were calculated for each growth stage. Correlation analysis was conducted between these VIs and SPAD values across different developmental stages (Figure 3). All indices showed positive correlations with SPAD values except NPCI. At the mid-grain-filling stage, the correlation between NRI and SPAD reached a significant level (*p* < 0.05), while highly significant correlations (*p* < 0.01) were observed between all other VIs and SPAD in the remaining growth stages. The VIs with the strongest correlation to SPAD varied across growth stages. OSAVI had the highest correlation coefficient of 0.74 with SPAD at the anthesis stage. In other stages, NDRE exhibited the strongest correlations, with coefficients of 0.65, 0.74 and 0.84 respectively. Considering the high correlation among various VIs, four VIs including NDRE, CIred-edge, GNDVI and OSAVI were selected to construct the GPC estimation model.

#### 2.2.2. Correlation Between Texture Information and SPAD Values

Correlation analysis was performed between extracted multispectral texture features and canopy SPAD values of wheat. At the jointing stage, 68.8% of the texture features showed highly significant correlations with SPAD values (*p* < 0.01). The Band4_VAR exhibited the highest correlation coefficient of −0.66, indicating a strong negative correlation. Correlations were markedly improved at the heading stage, where the proportion of highly significantly correlated features increased to 79.1%. Band4_ MEA had the strongest correlation with SPAD at this stage, with a coefficient of −0.73. By contrast, correlations decreased obviously at the anthesis stage, only 50.0% of texture features were highly significantly correlated with SPAD, and the maximum correlation coefficient was 0.66 for the Band5_MEA. At the mid-grain-filling stage, Band 4_VAR again had the strongest correlation with SPAD, with a coefficient of −0.71.

**Figure 4:**
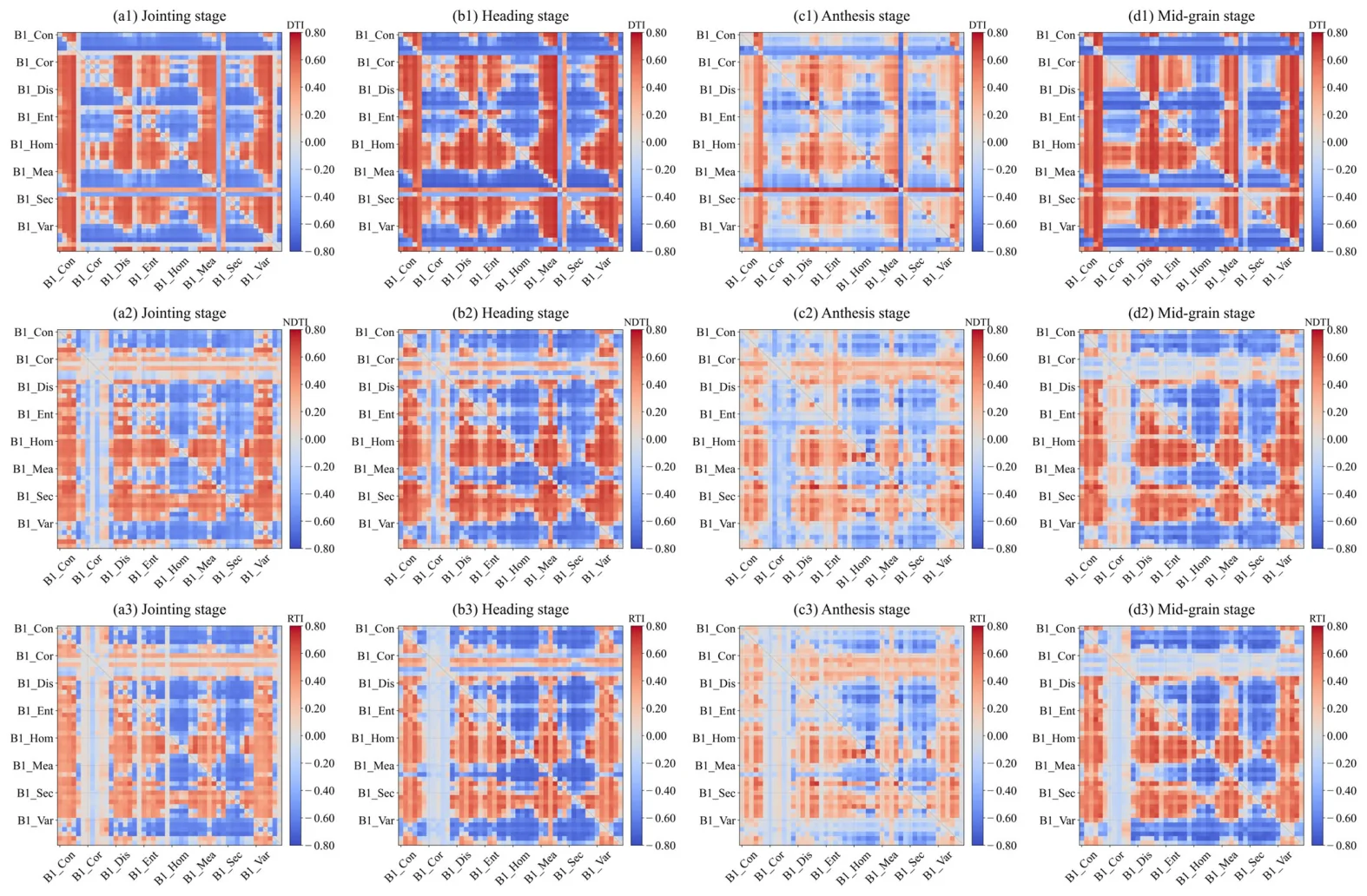
Pearson correlation coefficients of SPAD versus texture indices (TIs) of wheat at different growth stages.

TIs were constructed using different texture features, and their correlations with SPAD values were further analyzed. All TIs presented similar correlation trends with SPAD across all growth stages, with the most prominent correlations observed at the heading stage (Figure 4). Among the three types of TIs, difference texture index (DTI) had the highest overall correlation with SPAD, followed by normalized difference texture index (NDTI), while ratio texture index (RTI) showed relatively weak correlations. Sensitive TIs were established by selecting the optimal band combinations highly correlated with SPAD, as shown in Table 2. After screening, the correlations between the new sensitive TIs and SPAD were improved to varying degrees at each growth stage. The most notable improvement occurred at the anthesis stage, where the correlation coefficient increased to above 0.72. In terms of feature types, the texture indices sensitive to SPAD prediction were mainly constructed using the mean and variance derived from red-edge and near-infrared bands.

#### 2.2.3. Comparative Analysis of SPAD Modeling Results Based on Different Feature Fusion

In this study, leaf SPAD values exhibited an extremely significant and positive correlation with GPC at all key growth stages of wheat. Given this relationship, SPAD was adopted as a linking parameter between remote-sensing information and GPC to establish remote-sensing monitoring models for GPC. Accordingly, we first constructed a remote-sensing model for SPAD estimation. Using spectral reflectance of each band, VIs and optimal TIs as feature parameters, we evaluated the performance of different feature combinations for wheat SPAD modeling.

**Figure 5:**
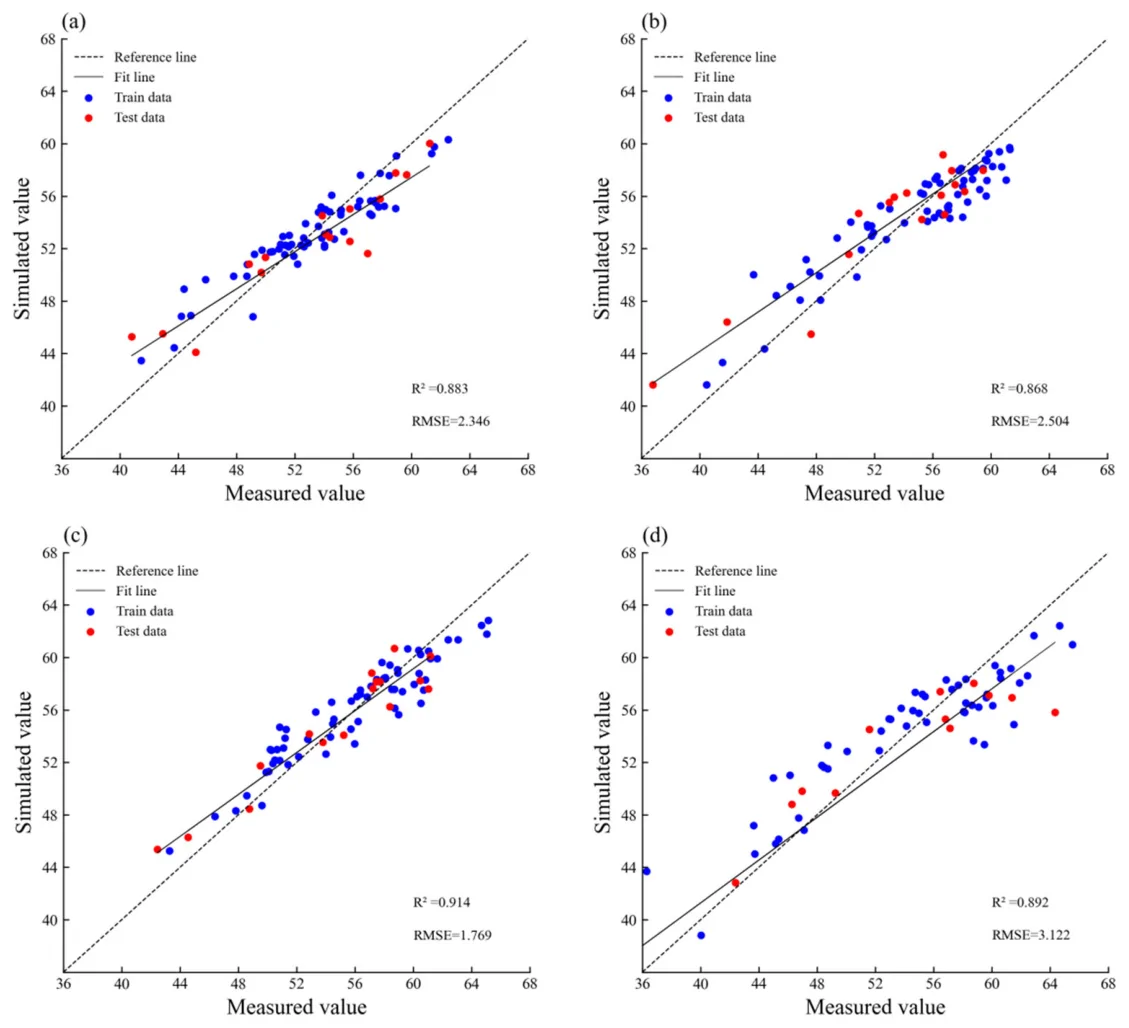
SPAD monitoring models for wheat canopy at different growth stages based on the optimal feature combination. (**a**) Jointing stage; (**b**) heading stage; (**c**) anthesis stage; and (**d**) mid-grain stage.

The inversion results (Table 3) showed that *R*^2^ of wheat SPAD models constructed solely with spectral reflectance were all higher than 0.80, with the RMSE below 3.7. Such models could effectively explain most variations in wheat SPAD values. Compared with models using single spectral reflectance, those based on VIs exhibited higher spectral sensitivity to SPAD at the heading and mid-grain-filling stage, with *R*^2^ increased by 0.02 and 0.06, respectively. In contrast, model accuracy slightly decreased at the jointing and anthesis stages, which was probably attributed to the selection of VIs. Nevertheless, the *R*^2^ remained above 0.8 and RMSE was less than 2.5 for both two stages. Further fusion of texture information and VIs improved the inversion accuracy. The *R*^2^ values increased by 0.07, 0.03 and 0.08 at the jointing, heading and anthesis stages, respectively, while the RMSE decreased by an average of 10% across all growth stages. The most remarkable improvement was observed at the anthesis stage, with *R*^2^ rising by 0.08 and RMSE declining by 0.46. However, the model accuracy decreased to a certain extent when band reflectance was further incorporated. In summary, the SPAD remote-sensing model integrating VIs and TIs achieved the best inversion performance. The anthesis stage obtained the highest modeling accuracy, with an *R*^2^ of 0.91 and an RMSE of 1.77 (Figure 5).

**Figure 6:**
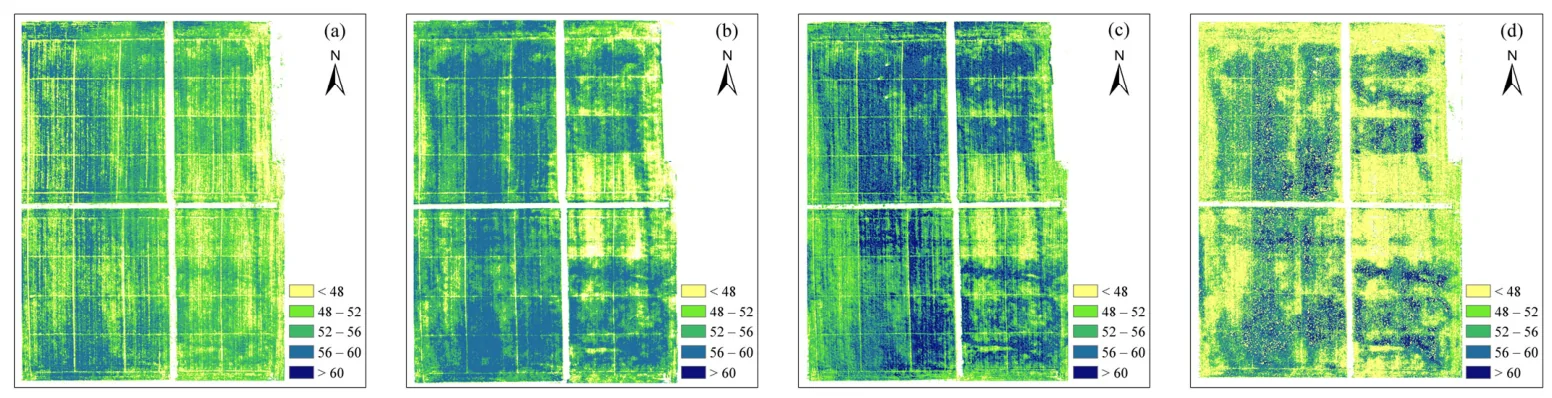
Spatial distribution of SPAD estimates at different growth stages based on unmanned aerial vehicle (UAV) imagery. (**a**) Jointing stage; (**b**) heading stage; (**c**) anthesis stage; and (**d**) mid-grain stage.

Accordingly, the Random Forest model combining VIs and TIs was adopted for spatial inversion of SPAD in winter wheat across four key growth stages. Figure 6 shows field-scale SPAD inversion results. The mean SPAD values across the four stages were 52.2, 53.9, 54.6 and 49.0. The results indicated that SPAD values increased from the jointing stage to the anthesis stage and then decreased obviously at the mid-grain-filling stage, which was basically consistent with field measurements. In terms of spatial distribution, winter wheat under high nitrogen treatment maintained relatively high SPAD levels.

Furthermore, the model was validated using field observations collected at the anthesis stage in 2024. The validation yielded a correlation coefficient of 0.80 and an RMSE of 3.83, which fully demonstrated the favorable generalization ability and practical application potential of the model.

### 2.3. Field-Scale GPC Models Based on Spectral Parameters and SPAD

Based on UAV-retrieved SPAD results, we extracted SPAD values within a 10 × 10 grid centered at the two sampling points of each field, and took the average as the SPAD value of the corresponding quadrat. Using SPAD as an intermediate parameter, this study established UAV-based wheat GPC estimation models by linear, exponential and polynomial methods. Modeling results for all growth stages showed that the model accuracy decreased in the order of polynomial model, exponential model and linear model, indicating a good nonlinear relationship between GPC and SPAD (Table 4). A comparison of the optimal models across growth stages revealed that *R*^2^ was approximately 0.60 and RMSE was around 1.1% for the jointing, heading and anthesis stages, with the anthesis-stage model achieving the highest accuracy. Due to the high dispersion of sampling data collected during the middle grain-filling stage of wheat, the *R*^2^ of its model was only 0.43, leading to unsatisfactory modeling performance. Therefore, the anthesis-stage model will be preferentially selected for subsequent regional-scale model construction.

**Figure 7:**
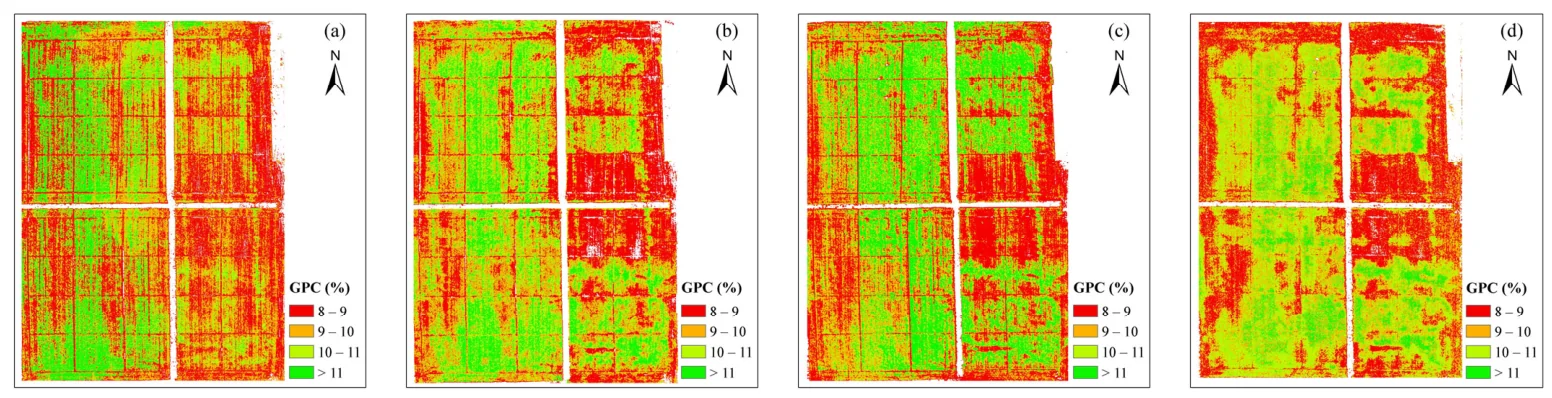
Spatial distribution of GPC estimates at different growth stages based on UAV. (**a**) Jointing stage; (**b**) heading stage; (**c**) anthesis stage; and (**d**) mid-grain stage.

The GPC estimation results for each growth stage are presented in Figure 7. The four mean values (9.6%, 9.7%, 9.9%, and 9.7%) were close to the measured value of 10.0%. At the anthesis stage, estimated GPC was 8.1–12.9% and measured GPC was 7.38–14.82%. Despite the tendency to overestimate low values and underestimate high values, the model performed well in estimating average GPC.

### 2.4. Retrieval of Wheat GPC at Satellite Scale

At the field scale, the model established with SPAD and GPC data at the wheat anthesis stage achieved the highest accuracy. Therefore, the datasets of this stage were selected to construct the wheat GPC retrieval model at the satellite scale.

The UAV-retrieved SPAD values of wheat at the anthesis stage were resampled to a spatial resolution of 10 m, consistent with that of Sentinel-2 imagery. The established quantitative model was adopted to calculate wheat GPC, and the results were matched pixel by pixel with the band reflectance and VIs derived from Sentinel-2 imagery. Based on Sentinel-2 multispectral data, a series of sensitive VIs, including RVI, NDRE, NPCI, NRI, CIred-edge, GNDVI and OSAVI, were calculated. Table 5 presents the correlation between band reflectance, VIs and GPC. The blue, green, red and red-edge 705 nm band reflectance showed negative correlations with UAV-retrieved SPAD, while the remaining bands were positively correlated. Among the VIs, RVI, NDRE1, NDRE2, GNDVI, CIred-edge1, CIred-edge2, and OSAVI all exhibited an extremely significant correlation with GPC (*p* < 0.01). Remote-sensing parameters with significant correlations to GPC were selected for model development.

**Figure 8:**
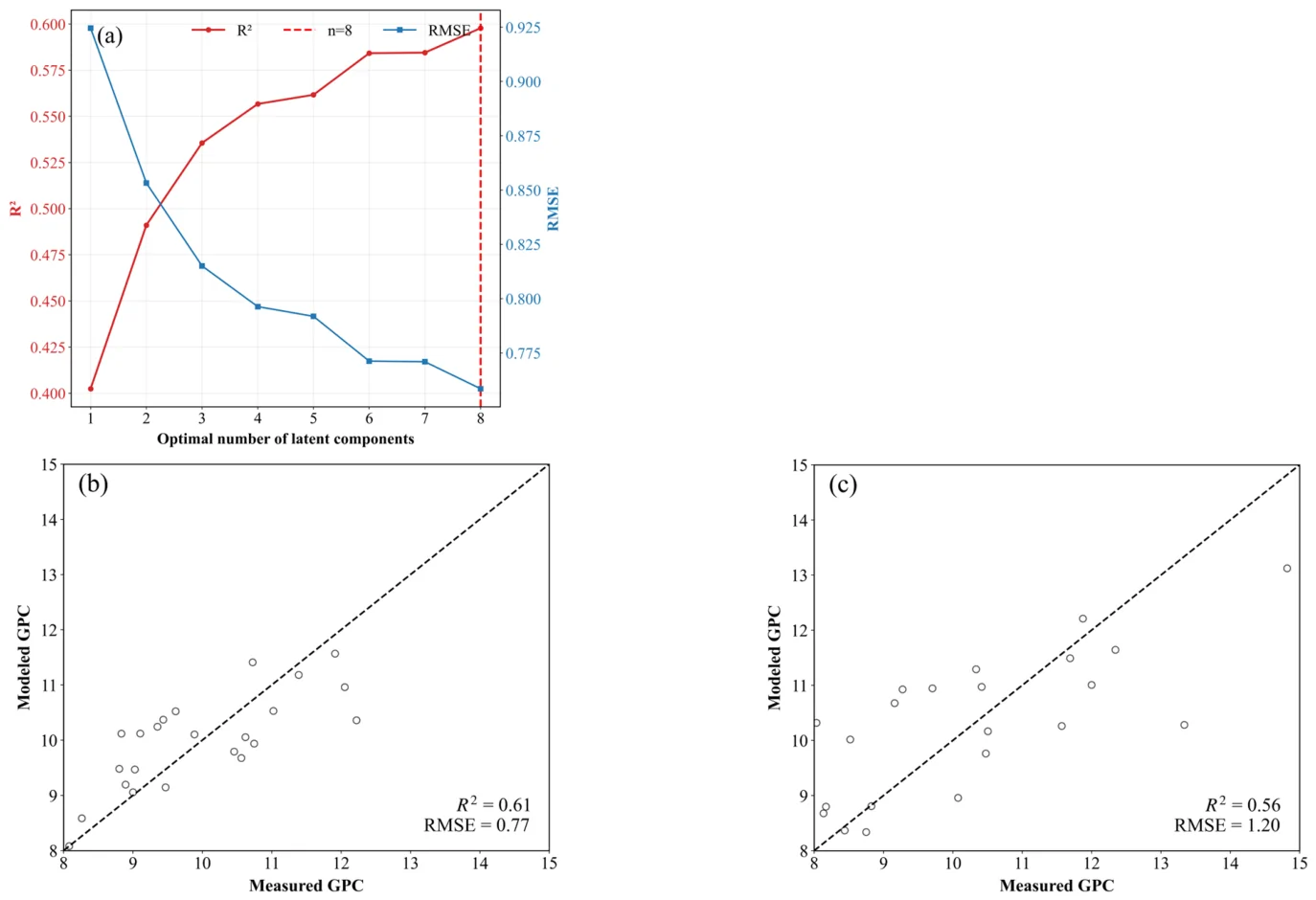
(**a**) Comparison of model accuracy with different feature parameters; (**b**) wheat GPC estimation model based on UAV upscaling data, where open circles denote paired modelled and measured GPC observations and the dashed line represents the 1:1 reference line; and (**c**) wheat GPC estimation model based on ground observation data.

**Figure 9:**
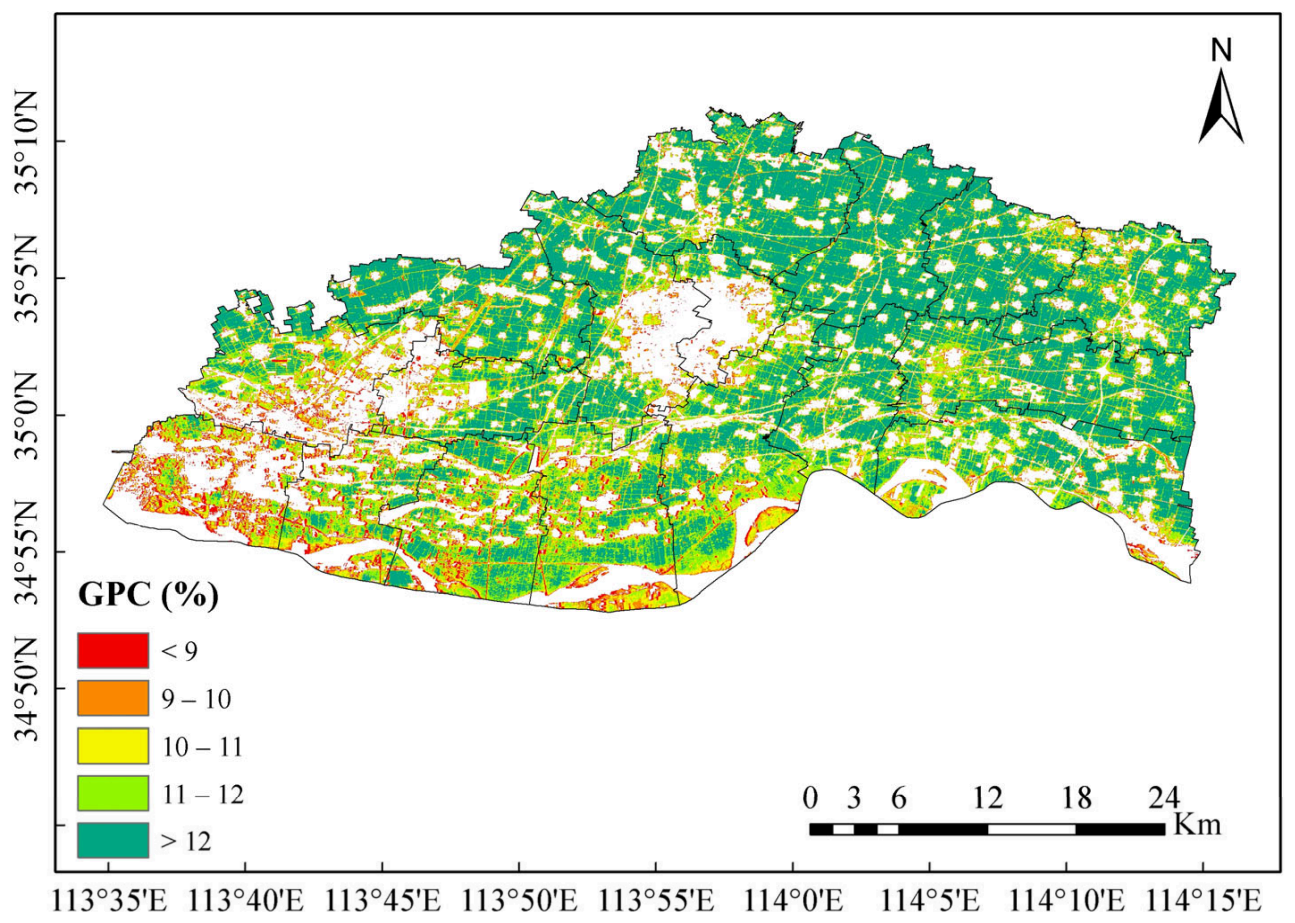
Estimation of wheat GPC in Xinxiang City, Henan Province, 2025.

To improve the regional stability and generalization performance of the GPC inversion model, the partial least squares regression (PLSR) method was adopted in this study to establish the estimation model. Prior to modeling, highly collinear features were eliminated by removing variables with correlation coefficients greater than 0.9. After screening, eight features were retained, namely B2, B3, B4, B5, B7, B8, NDRE2 and CIred-edge1 (Figure 8a). The results show that the model accuracy gradually increases with the rising number of principal components; the *R*^2^ rose by 48.5%, while the RMSE decreased by 18.0%. Considering both model complexity and simulation performance, eight parameters were finally selected to construct the model, with an *R*^2^ of 0.60 and an RMSE of 0.76. Compared with the model without scale conversion, the model built on UAV upscaled data achieved an 8.9% higher *R*^2^ and a 40% lower RMSE, demonstrating improved inversion accuracy (Figure 8b,c). The regional-scale estimation model of wheat GPC is given in Equation (1). Spatial estimation of wheat GPC over Xinxiang City in 2025 was carried out using this model, with the spatial distribution illustrated in Figure 9.

(1) GPC=5.077905−0.314459×B2+0.509426×B3−0.111049×B4+4.368774×B5+1.232459×B7−3.552147×B8+0.949803×NDRE2+5.520765×CIred-edge1

## 3. Discussion

### 3.1. Role of Texture Information in Estimating Wheat SPAD and GPC

Texture information can reflect differences in crop growth status and facilitate precise monitoring of wheat GPC. According to Sarker and Nichol (2011), textural features were capable of extracting spatial information irrespective of image tone, which facilitated the identification of image targets and regions of interest [37]. Fu et al. [12] constructed the NDVI_T_ by combining spectral and texture features, which substantially improved GPC estimation accuracy. Compared with traditional models based solely on VIs, the *R*^2^ of calibration and validation sets increased by 10% and 8%, respectively. This finding was consistent with the results reported by Chen et al. [38], further demonstrating the vital role of texture information in GPC monitoring. Nevertheless, texture parameters were not recommended for independent use due to their limited performance [39]. In this study, texture information was integrated into vegetation index-based models. After fusion, the *R*^2^ of validation sets increased by 8.6%, 3.6% and 9.6% at the jointing, heading and anthesis stages respectively, while the RMSE decreased across all four growth stages, with a maximum reduction of 20.1% at the anthesis stage. Overall, texture information serves as a valuable auxiliary variable and can achieve better predictive performance when combined with spectral data.

### 3.2. Potential of Multi-Source Data Fusion for Estimating Wheat SPAD and GPC

Fine-scale crop monitoring is easily achieved at the field scale, whereas high precision monitoring and assessment over large regional scales remains a challenge. Satellite remote-sensing serves as a core tool for large-scale crop monitoring. However, scale effects between field measured data and satellite imagery greatly limit monitoring accuracy. Existing studies have adopted UAV remote-sensing data as a bridge between ground measurements and satellite data, which effectively alleviates the scale mismatch of multi-source data and enables refined monitoring of crop growth, yield, and quality at regional scales [40,41].

Yuan et al. [42] calibrated Sentinel-2 and Sentinel-3 satellite imagery using the mean ratio and temperature deviation methods to achieve radiometric consistency, increasing the *R*^2^ of the maize plant moisture content estimation model by 0.12–0.19. Fathololoumi et al. [20] scaled UAV-derived yield data to the 10 m grid of Sentinel-2 imagery, improved the accuracy of corn yield prediction, and reduced the prediction uncertainty across multiple spatial scales, with the proportion of high-uncertainty areas in farmland decreasing by an average of 44.1%. Similarly, in this study, UAV-inverted wheat GPC results were resampled to a 10 m resolution to construct a regional-scale GPC estimation model. The results showed that compared with the model without scale conversion, the model constructed based on UAV upscaled data improved the *R*^2^ by 8.9% and reduced the RMSE by 40%. The proposed approach outperformed the study by Liu et al. [18], who merely integrated UAV-derived features for regional-scale model construction. Nevertheless, although such data fusion methods can effectively improve the inversion accuracy of regional models, the upscaling process mainly relies on spatial averaging or statistical interpolation, which fails to eliminate differences in the spectral response functions of sensors across different remote-sensing platforms and thus has certain application limitations [43,44].

The regional inversion model constructed in this study only adopted two types of features, resulting in a relatively single feature dimension. Previous studies have confirmed that integrating multi-dimensional remote-sensing features such as water indices and temperature parameters can further optimize model performance [45,46]. In addition, model validation in this study was conducted only based on local datasets without cross-regional sample verification, so the spatial generalization ability of the model needs to be further verified and improved.

### 3.3. Limitations of GPC Estimation Models

A quantitative estimation model for field-scale wheat GPC was constructed by integrating remote-sensing information and agronomic parameters, with reference to the advantages and limitations of existing models. Correlation analysis revealed that the GPC of mature wheat exhibited significant positive correlations with SPAD values measured at all growth stages; the correlation coefficient peaked at the anthesis stage, showing a stronger correlation than that between LAI and GPC. Accordingly, SPAD was selected as the linking parameter connecting spectral features and wheat grain protein content. The estimation model constructed based on the anthesis stage achieved optimal accuracy, with the *R*^2^ of 0.61 and RMSE of 1.11%. Its simulation results could adequately reflect the wheat GPC level of each field plot; nevertheless, the model suffered from systematic bias consisting of the underestimation of high values and overestimation of low values, which was mainly attributed to the limited sample size and single modeling algorithm adopted in this study. Only 28 groups of measured GPC data were collected after wheat ripening and harvesting, and the insufficient sample size restricted the generalization performance of the model. In this study, the Random Forest algorithm was first adopted to establish the SPAD inversion model, followed by polynomial regression for GPC estimation, without comparative screening among multiple machine learning algorithms. Existing studies verified that machine learning algorithms, including partial least squares regression, artificial neural networks and support vector machines, performed well in remote-sensing estimation of wheat nutritional indicators. However, machine learning models acted as black-box models and were slightly less practical than empirical models [47].

In addition, wheat cultivars inherently differ in nitrogen uptake efficiency [48,49]. Data analysis in this study revealed only slight differences in GPC between the two tested wheat varieties. The main wheat cultivars planted in Yuanyang County and the wider Xinxiang city mainly include the Zhengmai, Zhoumai and Bainong series, which belong to medium-gluten and medium-strong-gluten wheat with relatively consistent varietal characteristics. Therefore, the model established in this study is applicable to local dominant wheat varieties and can be reliably used for regional GPC estimation. Nevertheless, genotype is still a crucial internal factor determining winter wheat GPC. Future modeling studies should fully consider varietal differences and incorporate genotypic information to further improve the universality and estimation accuracy of the model.

Field environmental conditions constituted one of the critical factors affecting high yield and superior grain quality of wheat [50,51]. Meteorological conditions characterized by drought, scarce rainfall and sufficient solar radiation generally facilitated the improvement of wheat GPC [52]. Wang et al. [53] further analyzed the effects of meteorological conditions on wheat GPC based on remote-sensing data and key agronomic parameters; the *R*^2^ of GPC model increased by 0.242 compared with the model without meteorological variables incorporated. This study lacked multi-year and multi-field observational data, so meteorological factors were not taken into account when constructing the GPC estimation model. The simulation results were susceptible to seasonal characteristics, planting environments and experimental regions, and the application of this model to other environments would lead to unsatisfactory GPC estimation accuracy.

Furthermore, this study found that the model accuracy for the mid-filling stage was considerably lower than that for the jointing, heading, and anthesis stages. This phenomenon was mainly attributed to the distinct differences in plant senescence processes and SPAD values under varied nitrogen treatments. Under low nitrogen supply, the SPAD values declined rapidly. In contrast, adequate nitrogen application maintained relatively high leaf SPAD levels. Such obvious differentiation in crop physiological status across different nitrogen regimes disrupted the stable coupling relationship between SPAD values and grain protein content during the filling period, thereby reducing the model estimation accuracy at this stage.

## 4. Materials and Methods

### 4.1. Experimental Design

**Figure 10:**
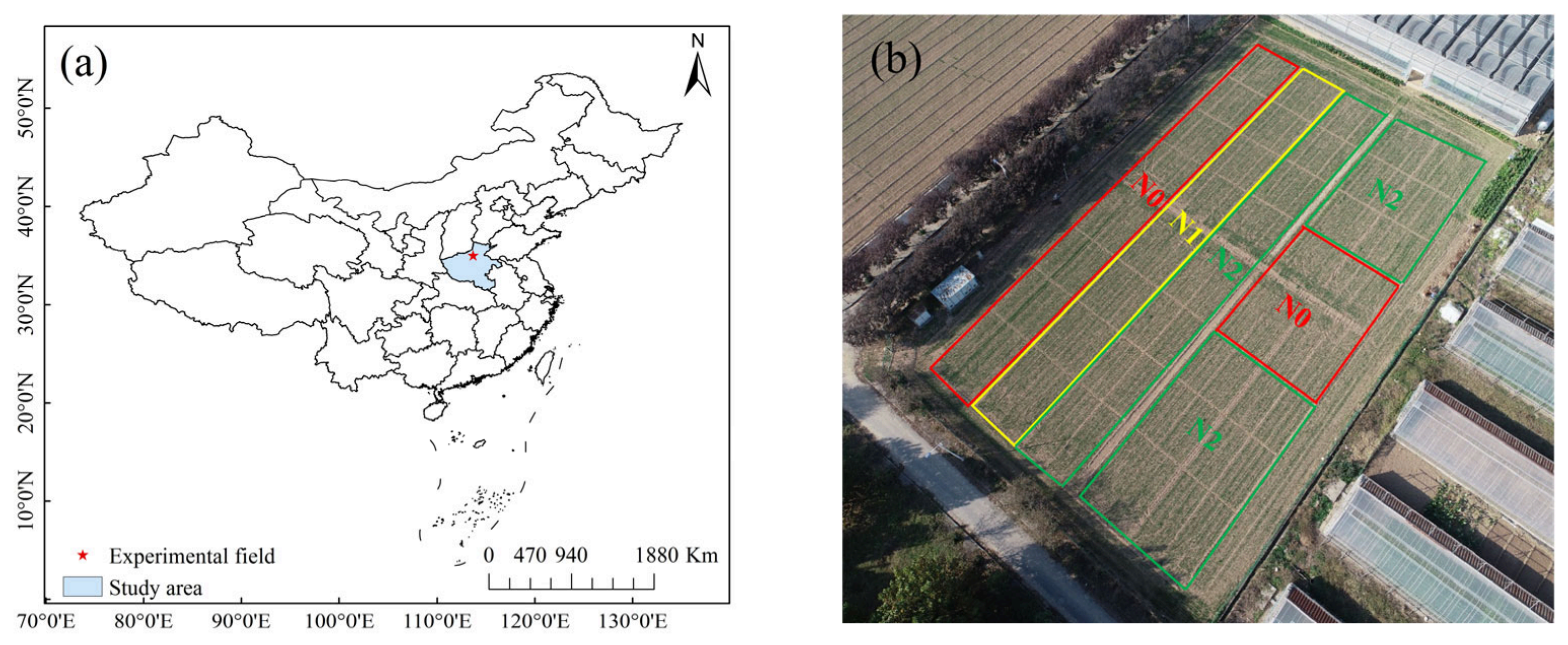
(**a**) Location of study area; (**b**) experimental design. N0, N1 and N2 represent nitrogen application levels of 90 kg N ha^−1^, 180 kg N ha^−1^, and 270 kg N ha^−1^, respectively.

The field experiment was conducted across two consecutive growing seasons (2023–2024 and 2024–2025) in Yuanyang, Xinxiang, Henan Province (35°00′ N, 113°41′ E, Figure 10a). As a national demonstration zone for green, high-quality, and efficient wheat production, the region has fertile soils and favorable irrigation conditions. The local winter wheat growing period ranges from early October to early June of the following year. The study area has a warm temperate continental monsoon climate with four distinct seasons. Its long-term mean annual temperature is 14.4 °C, mean annual precipitation is 550–600 mm, and the frost-free period is approximately 227 days. For the 2023–2024 and 2024–2025 growing seasons, the study area recorded mean air temperatures of 12.7 °C and 13.2 °C, pre-winter accumulated temperatures of 440.7 °C·d and 347.2 °C·d, total precipitation values were 296.2 mm and 314.5 mm, and sunshine durations were 1637.6 h and 1649.4 h, respectively. Both growing seasons had sufficient light and heat resources as well as suitable soil moisture. Meteorological disasters were generally mild, which was favorable for winter wheat growth.

A total of 40 independent experimental plots were established, each covering an area of 8 m × 8 m (Figure 10b). Two dominant local wheat cultivars, *Triticum aestivum* L. cv. Zhengmai 136 and *Triticum aestivum* L. cv. Zhoumai 36, were planted in the northern and southern parts of the experimental field, respectively. A uniform plant density of 300 × 10^4^ plants ha^−1^ was applied across all plots. Three nitrogen application levels were set at 90 kg N ha^−1^ (N0), 180 kg N ha^−1^ (N1), and 270 kg N ha^−1^ (N2). A basal nitrogen fertilizer of 90 kg N ha^−1^ was applied before sowing, and the remaining nitrogen was topdressed at the jointing stage. According to the FAO soil classification system, the experimental site had clay-loam soil. Selected physicochemical properties of the 0–20 cm topsoil layer were as follows: pH 7.7, organic matter of 11.2 g kg^−1^, total nitrogen of 0.8 g kg^−1^, Olsen-P of 23.4 mg kg^−1^, and available potassium of 171.5 mg kg^−1^. Supplemental irrigation was carried out by micro-sprinkler irrigation according to soil moisture conditions to maintain suitable soil water status for wheat growth. All other field management followed conventional management for high-yield wheat production, ensuring uniformity of non-experimental variables.

### 4.2. Data Acquisition and Processing

Field spectral measurement and sample collection were carried out on 26 April 2024 (anthesis stage), as well as 21 March (jointing stage), 2 April (heading stage), 14 April (anthesis stage), and 9 May (mid-grain-filling stage) in 2025. Within each plot, representative, uniform and disease-free 1 m × 1 m sampling quadrats were established. Two 1 m measuring rods were placed in each quadrat.

#### 4.2.1. Ground Observation Data

The LAI of winter wheat was measured using a LAI-2200C plant canopy analyzer (LI-COR Biosciences, Lincoln, NE, USA). During measurements, the lens was maintained in a horizontal position while direct solar radiation was avoided as much as possible. With the operator facing away from the sun, one above-canopy reading was first collected, followed by four sequential below-canopy readings taken by placing the analyzer close to the root base of winter wheat plants. Each sampling point was measured in the three replicates, and the average value was calculated as the LAI of the point. Leaves’ relative chlorophyll content, denoted as SPAD values, was determined using a SPAD-502 chlorophyll meter (Konica Minolta, Osaka, Japan). Healthy and representative winter wheat leaves at each growth stage were selected for measurement, with care taken to avoid leaf overlap during testing. Five replicates were measured at each sampling point, and the average value was used to represent the chlorophyll content. In addition, the latitude and longitude of sampling points were recorded. At the wheat maturity stage, plants within each 1 m × 1 m quadrat were harvested. After threshing and natural air-drying, GPC was determined by using a near-infrared grain analyzer and the Kjeldahl method respectively.

**Figure 11:**
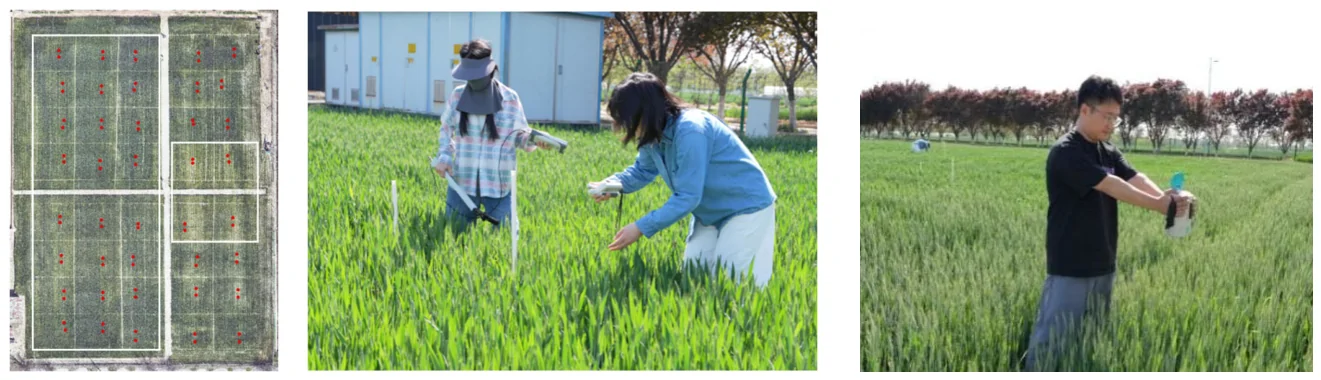
Distribution of sampling points and field data collections. Red dots represent sampling points for SPAD and LAI; plots marked with white boxes were sampled for GPC.

In 2024, field sampling was conducted in 24 plots on the western side of the experimental area, yielding 24 sets of observational data. To increase the sample size, the sampling scope was expanded in 2025. The distribution of ground-sampling points is shown in Figure 11. Two sampling points were arranged within each plot for LAI and SPAD, while plots marked with white boxes were sampled for GPC. In 2025, a total of 80 paired LAI–SPAD observations and 28 grain samples for GPC analysis were acquired. Data collected in 2025 were used for model development. The complete 2024 dataset was used for cross-year independent validation to assess the model’s generalization capability across growing seasons.

#### 4.2.2. UAV Multispectral Data

In this study, a DJI M300 RTK UAV (DJI, Shenzhen, China) equipped with an Altum-PT multispectral camera (MicaSense, Seattle, WA, USA) was utilized. The camera covered six spectral bands, including blue (475.0 ± 16.0 nm), green (560.0 ± 13.5 nm), red (668.0 ± 7.0 nm), red-edge (717.0 ± 6.0 nm), near-infrared (842.0 ± 28.5 nm), and thermal infrared (7.5–13.5 μm) bands. This system was capable of synchronously acquiring RGB image, thermal maps and high-resolution panchromatic data. Wheat canopy spectral data were collected between 10:00 and 14:00 under cloudless conditions with a relatively stable atmospheric environment. The flight route was predefined using the DJI Pilot application (V2.5.1.15). To ensure high image resolution and stitching quality, both the forward and side overlap rates were set to 80% with a flight altitude of 60 m, and absolute reflectance radiometric calibration was conducted using the MicaSense Calibrated Reflectance Panel prior to each flight mission. Agisoft Metashape Professional software (version 2.0.0) was adopted for multispectral image mosaicking and post-processing, generating orthomosaic images with a pixel resolution of 5 cm. To match the ground sampling data, we extracted the spectral reflectance of pixels within the 10 × 10 grid window surrounding each ground sampling point for sensitive parameter screening and model construction.

#### 4.2.3. Satellite Data

Preprocessed Sentinel-2 Level-2A remote-sensing datasets were obtained from the AI Earth Geoscience Cloud Platform (https://engine-aiearth.aliyun.com/#/ (accessed on 26 January 2026)), which had been processed with radiometric calibration, orthorectification, and atmospheric correction. The Sentinel-2 multispectral instrument comprised 13 spectral bands with spatial resolutions of 10, 20, and 60 m. Uniquely among operational satellites, Sentinel-2 carried three red-edge bands closely related to vegetation physicochemical parameters, making it highly effective for vegetation monitoring. In this paper, we used spectral bands at 10 and 20 m resolution, and the 20m bands were resampled to 10 m using ENVI software (version 5.6). Combined with the UAV flight experiments in the field, we selected Sentinel-2 images acquired on 26 April 2024 and 16 April 2025 for satellite-scale SPAD inversion and validation, respectively. 

### 4.3. Spectral Parameters

#### 4.3.1. VIs

VIs were derived from spectral reflectance of different bands, which could better reflect vegetation growth conditions than raw spectral bands. Wheat GPC was closely related to nitrogen nutrition, and changes in nitrogen availability directly alter leaf chlorophyll content. Therefore, VIs highly correlated with chlorophyll and nitrogen contents were screened to establish inversion models. Previous studies showed that RVI presented optimal accuracy for wheat GPC estimation [54]. Main et al. [55] analyzed 73 typical VIs and demonstrated that red-edge-based VIs served as effective indicators for wheat canopy nitrogen status. Additionally, three-band VIs showed advantages in GPC monitoring, as they integrated abundant spectral information and improved the interpretation capability of complex canopy structures [56]. Accordingly, seven indices including RVI, NDRE, NPCI, NRI, CIred-edge, GNDVI and OSAVI were selected for subsequent analysis, as shown in Table 6.

#### 4.3.2. TIs

Image texture denotes visual features of homogeneous surface patterns, representing slowly varying or periodically arranged structural characteristics on object surfaces. As complements to spectral information and VIs, texture features can effectively monitor key crop growth parameters, including chlorophyll content, above-ground biomass and LAI. In this study, texture features were extracted via the second-order probability statistical filtering method. A 3 × 3 sliding window with a moving step of 1 and 64-level gray quantization was adopted. Eight types of texture features were derived from different spectral bands, namely Mean (MEA), Variance (VAR), Homogeneity (HOM), Contrast (CON), Dissimilarity (DIS), Entropy (ENT), Second Moment (SEC) and Correlation (COR). Three combined TIs, including DTI, NDTI and RTI, were further established by pairwise combination of the above texture features.

(2) $${\mathrm{DTI}}_{\left(i,j\right)}={\mathrm{TF}}_{\left(i\right)}-{\mathrm{TF}}_{\left(j\right)},$$

(3) $${\mathrm{NDTI}}_{\left(i,j\right)}=\frac{{\mathrm{TF}}_{\left(i\right)}-{\mathrm{TF}}_{\left(j\right)}}{{\mathrm{TF}}_{\left(i\right)}+{\mathrm{TF}}_{\left(j\right)}},$$

(4) $${\mathrm{RTI}}_{\left(i,j\right)}=\frac{{\mathrm{TF}}_{\left(i\right)}}{{\mathrm{TF}}_{\left(j\right)}},$$

where ${\mathrm{TF}}_{\left(i\right)}$ and ${\mathrm{TF}}_{\left(j\right)}$ represent the *i*-th and *j*-th texture features, respectively.

### 4.4. Model Construction

With the rapid growth of spaceborne and airborne imaging spectrometers, the era of big data in remote-sensing has arrived. Machine learning, with its ability to handle complex nonlinear problems, can effectively utilize a large number of canopy spectral features to simulate wheat plant nitrogen content and predict GPC.

The Random Forest algorithm was adopted to establish the estimation model for field-scale SPAD. As a classic parallel ensemble learning method, it integrates prediction results from multiple decision trees to achieve high estimation precision. Each decision tree remains interpretable, while random feature sampling reduces the model’s sensitivity to training data and mitigates overfitting, enabling the model to capture both linear and nonlinear relationships. In modeling, 80% of samples acquired in 2025 were randomly assigned to the training set, and the remaining 20% were used for internal validation. Key parameters including maximum iteration number and tree depth were optimized to achieve optimal model performance, while other parameters remained at default. Meanwhile, linear, exponential and polynomial methods were used to construct UAV-based estimation models for wheat GPC. The ground samples of 2024 were applied for cross-year validation.

In terms of regional GPC, we used the PLSR to enhance the regional applicability and stability of the inversion model. PLSR is applied to quantify the relationships between multiple independent variables and dependent variables. It integrates the fundamental functions of multiple linear regression and principal component analysis. It does not require independent variables to be mutually independent, and can eliminate the interference caused by multicollinearity among independent variables on model accuracy. It achieves optimal fitting performance by searching for the optimal dimensionality-reduction subspace.

RMSE and *R*^2^ were employed to evaluate model performance.

## 5. Conclusions

In this study, multi-source data from satellites, UAVs and ground surveys were integrated to establish remote-sensing estimation models for wheat GPC suitable for various wheat growth stages and multiple scales. Based on field experiments, the relationship between wheat physiological parameters and grain quality was clarified, and sensitive remote-sensing indices were selected to establish a reliable estimation framework for accurate and dynamic GPC inversion. At the field scale, the GPC estimation model for the anthesis stage exhibited the best comprehensive performance. Cross-year validation using independent field data collected in 2024 achieved an RMSE of 1.01, indicating good model stability and generalization capability. Through scale transformation, UAV-retrieved GPC data were upscaled to a 10 m resolution, and a regional-scale GPC estimation model was further constructed using Sentinel-2 satellite imagery, realizing the effective expansion of wheat quality monitoring from field to regional scales.

The proposed multi-scale remote-sensing method overcomes the drawbacks of traditional single-point monitoring and resolves the scale mismatch between ground observation data and satellite remote-sensing data. It provides a novel technical approach for dynamic monitoring of wheat quality and offers scientific support for the management and decision-making of agricultural production. Nevertheless, this study has certain limitations. The regional models were constructed without incorporating meteorological covariates, and model calibration was performed using datasets from only a limited number of experimental years. Accordingly, the spatial-temporal generalization capacity of the established models under diverse climatic conditions, planting patterns, and wheat cultivars require further verification. Future research will deepen the application of hyperspectral UAV remote-sensing data and conduct model construction and validation across multiple regions. Meanwhile, multiple influencing variables such as meteorological factors will be incorporated to improve the generalization ability and estimation accuracy of the model under complex environmental conditions, so as to continuously upgrade the application performance of the remote-sensing monitoring technical system for wheat quality.

## Figures and Tables

**Table 1:** Pearson correlation coefficients of grain protein content (GPC) versus SPAD and LAI at different growth stages.

Growth Stages	SPAD	LAI
Jointing stage	0.73 **	0.33
Heading stage	0.71 **	0.72 **
Anthesis stage	0.74 **	0.65 **
Mid-grain stage	0.62 **	0.41 *

Note: * and ** indicate significance at the 0.05 and 0.01 probability levels, respectively.

**Table 2:** Pearson correlation coefficients of optimal TIs versus SPAD.

Growth Stages	Optimal Combination	Correlation Coefficient
Jointing stage	DTI (Band6_ENT, Band4_VAR)	0.67 **
	NDTI (Band5_MEA, Band4_VAR)	0.66 **
	RTI (Band4_HOM, Band5_SEC)	0.63 **
Heading stage	DTI (Band1_MEA, Band4_MEA)	0.75 **
	NDTI (Band5_MEA, Band4_MEA)	0.74 **
	RTI (Band2_HOM, Band6_HOM)	0.72 **
Anthesis stage	DTI (Band5_MEA, Band4_MEA)	0.77 **
	NDTI (Band5_MEA, Band5_DIS)	0.75 **
	RTI (Band5_MEA, Band5_DIS)	0.72 **
Mid-grain stage	DTI (Band1_MEA, Band2_MEA)	0.74 **
	NDTI (Band3_ENT, Band4_VAR)	0.73 **
	RTI (Band3_ENT, Band4_VAR)	0.69 **

Note: ** indicate significance at the 0.01 probability levels.

**Table 3:** Comparison of SPAD inversion accuracy using Random Forest regression models with different feature combinations.

Growth Stages	Feature Combinations	*R* ^2^	RMSE
Jointing stage	REFs	0.81	2.46
	VIs	0.81	2.48
	VIs + TIs	0.88	2.36
	VIs + TIs + REFs	0.87	2.41
Heading stage	REFs	0.82	2.34
	VIs	0.84	2.59
	VIs + TIs	0.87	2.50
	VIs + TIs + REFs	0.87	2.61
Anthesis stage	REFs	0.88	1.52
	VIs	0.83	2.23
	VIs + TIs	0.91	1.77
	VIs + TIs + REFs	0.91	1.83
Mid-grain stage	REFs	0.86	3.68
	VIs	0.92	3.39
	VIs + TIs	0.89	3.12
	VIs + TIs + REFs	0.87	3.64

Note: REFs, multiple-band spectral reflectance; VIs, selected vegetation indices including NDRE, CIred-edge, GNDVI, and OSAVI; and TIs, selected optimal texture index combinations.

**Table 4:** UAV-based estimation models for wheat GPC using spectral parameters and SPAD.

Growth Stages	Models	Formulas	*R* ^2^	RMSE (%)
Jointing stage	Linear	y = 0.268x − 4.3727	0.53	1.21
	Exponential	y = 2.3743e^0.0265x^	0.57	1.18
	Polynomial	y = 0.0145x^2^ − 1.2365x + 34.418	0.60	1.12
Heading stage	Linear	y = 0.22x − 1.8482	0.50	1.26
	Exponential	y = 2.9931e^0.0221x^	0.55	1.23
	Polynomial	y = 0.0161x^2^ − 1.4182x + 39.085	0.59	1.14
Anthesis stage	Linear	y = 0.2762x − 5.099	0.55	1.19
	Exponential	y = 2.2582e^0.0269x^	0.57	1.16
	Polynomial	y = 0.0148x^2^ − 1.3132x + 37.196	0.61	1.11
Mid-grain stage	Linear	y = 0.0949x + 5.3034	0.38	1.39
	Exponential	y = 6.1235e^0.0096x^	0.43	1.38
	Polynomial	y = 0.0027x^2^ − 0.1344x + 9.672	0.43	1.37

**Table 5:** Pearson correlation coefficients between remote-sensing features and GPC.

Features	Correlation Coefficients	Features	Correlation Coefficients	Features	Correlation Coefficients
B2 (Blue)	−0.38 *	B8 (Near-infrared)	0.55 **	NRI	0.06
B3 (Green)	−0.41 *	RVI	0.53 **	GNDVI	0.52 **
B4 (Red)	−0.40 *	NDRE1	0.55 **	CIred-edge1	0.55 **
B5 (red-edge 705)	−0.36 *	NDRE2	0.55 **	CIred-edge2	0.55 **
B6 (red-edge 740)	0.24	NDRE3	0.02	CIred-edge3	0.02
B7 (red-edge 783)	0.55 **	NDRE4	0.14	CIred-edge4	−0.06
B8A (red-edge 865)	0.43 **	NPCI	−0.05	OSAVI	0.54 **

Note: * and ** indicate significance at the 0.05 and 0.01 probability levels, respectively. B2–B8 are reflectance bands of the Sentinel-2 satellite. B2, B3, and B4 correspond to the blue, green, and red bands, respectively; B5, B6, B7, and B8A are red-edge bands; B8 denotes the near-infrared band. NDRE1-NDRE4 are NDRE indices calculated from B5, B6, B7, and B8A in combination with the B8 band, respectively; CIred-edge1–CIred-edge4 are computed using the corresponding red-edge bands together with the B8 band.

**Table 6:** VIs related to monitoring wheat GPC.

VIs	Formulation
Ratio vegetation index (RVI)	$R_{\mathrm{nir}}/R_{\mathrm{red}}$
Normalized difference red-edge index (NDRE)	$\left(R_{\mathrm{nir}}-R_{\text{red\_edge}})/(R_{\mathrm{nir}}+R_{\text{red\_edge}}\right)$
Normalized pigments chlorophyll ratio index (NPCI)	$\left(R_{\mathrm{red}}-R_{\mathrm{blue}})/(R_{\mathrm{red}}+R_{\mathrm{blue}}\right)$
Nitrogen reflectance index (NRI)	$\left(R_{\mathrm{green}}-R_{\mathrm{red}})/(R_{\mathrm{green}}+R_{\mathrm{red}}\right)$
Red chlorophyll edge index (CIred-edge)	$R_{\mathrm{nir}}/R_{\text{red\_dege}}-1$
Green normalized difference vegetation index (GNDVI)	$\left(R_{\mathrm{nir}}-R_{\mathrm{green}})/(R_{\mathrm{nir}}+R_{\mathrm{green}}\right)$
Optimized soil-adjusted vegetation index (OSAVI)	$1.16(R_{\mathrm{nir}}-R_{\mathrm{red}})/(R_{\mathrm{nir}}+R_{\mathrm{red}}+0.16)$

## Data Availability

The data presented in this study are available on request from the corresponding author due to the ongoing nature of the research project, which involves further data collection and analysis that have not yet been completed.

## Abbreviations

The following abbreviations are used in this manuscript:

GPC	Grain protein content
UAV	Unmanned aerial vehicle
VIs	Vegetation indices
TIs	Texture indices
SPAD	Soil–Plant Analysis Development
RMSE	Root mean square error
LAI	Leaf area index
PLSR	Partial least squares regression
RVI	Ratio vegetation index
NDRE	Normalized difference red-edge index
NPCI	Normalized pigments chlorophyll ratio index
NRI	Nitrogen reflectance index
CIred-edge	Red chlorophyll edge index
GNDVI	Green normalized difference vegetation index
OSAVI	Optimized soil-adjusted vegetation index
DTI	Difference texture index
NDTI	Normalized difference texture index
RTI	Ratio texture index
